# Torsion and ruptured ovarian cystadenocarcinoma with internal bleeding complicated with retroperitoneal hematoma after tumor transection: A case report

**DOI:** 10.1097/MD.0000000000041282

**Published:** 2025-01-24

**Authors:** Chin-Tzu Tien, Chiu-Hsuan Cheng, Mun-Kun Hong

**Affiliations:** a Department of Obstetrics and Gynecology, Minimally Invasive Gynecology Surgery Center, Hualien Tzu Chi Hospital, Buddhist Tzu Chi Medical Foundation, Hualien, Taiwan; b Department of Anatomical Pathology, Hualien Tzu Chi Hospital, Buddhist Tzu Chi Medical Foundation, Hualien, Taiwan; c Institute of Medical Sciences, Tzu Chi University, Hualien, Taiwan; d School of Medicine, Tzu Chi University, Hualien, Taiwan, R.O.C.

**Keywords:** ovarian cancer, ovarian torsion, ruptured tumor

## Abstract

**Rationale:**

Ovarian tumor torsion is a critical gynecological emergency, predominantly affecting women of reproductive age, with benign teratomas being the most common culprits. In contrast, malignant ovarian tumors, such as mucinous cystadenocarcinoma, infrequently present with torsion due to their invasive and angiogenic characteristics. The occurrence of torsion in malignant tumors complicates diagnosis and management, particularly when associated with complications like congestion, infarction, and internal bleeding. This report details a rare case of primary ovarian mucinous cystadenocarcinoma presenting with acute torsion and significant internal bleeding. Our study highlights the diagnostic challenges and the urgent need for clear treatment guidelines, addressing an important gap in the existing literature regarding the management of torsion malignant ovarian tumors. By documenting this case, we aim to contribute to the understanding of this rare condition and provide insights that may help clinicians in similar scenarios.

**Patient concerns:**

A 51-year-old postmenopausal woman presented with acute abnormal pain. Transvaginal ultrasound examination showed an 8-cm heterogeneous right ovary mass without ovarian blood flow on color Doppler.

**Diagnoses:**

Laparoscopy revealed torsion and rupture of the right ovarian tumor with 900 mL hemoperitoneum. The patient underwent right salpingo-oophorectomy complicated by continuous oozing and hematoma formation in the infundibular ligament. Unexpectedly, histopathology revealed a mucinous cystadenocarcinoma of the right ovary, pT1c2.

**Intervention:**

The patient underwent staging surgery and prophylactic hyperthermic intraperitoneal chemotherapy.

**Outcome:**

After 4 years of follow-up, no tumor recurrence or metastasis was found.

**Lessons:**

Currently, there are no effective preoperative diagnostic and treatment guidelines for ruptured malignant ovarian tumors with torsion. The possibility of malignancy should be considered, and frozen section biopsy should be considered during surgery. Full detorsion before tumor resection to avoid incomplete pedicle coagulation and bleeding. Specimen removal by in-bag morcellation in minimally invasive surgery to prevent complications related to residual fragments of the specimen or dissemination of an occult malignancy.

## 1. Introduction

Adnexal torsion mainly occurs in women of reproductive age, especially in those with teratomas. Approximately 2% to 15% of patients who underwent surgical treatment of adnexal masses had ovarian torsion.^[1,2]^ Laparoscopy is an effective method of reducing the number of necessary laparotomies when the diagnosis of torsion is uncertain. Most ovarian tumors with torsion are predominantly benign, and the most common symptom of ovarian torsion/rupture is abrupt colicky pain in 1 lower quadrant of the abdomen, sometimes followed by nausea and vomiting.^[2]^ Patients with incomplete ovarian torsion may experience intermittent lower abdominal pain and asymptomatic symptoms. Preoperative diagnosis of ovarian torsion/rupture may be difficult because of the low sensitivity and specificity of clinical parameters, and the manifestation overlaps with symptoms from numerous other etiologies. Hibbard^[3]^ even reported that a few patients with minimal pelvic and abdominal pain were found to have ovarian torsion or infarction when an elective operation with a preoperative diagnosis of an ovarian tumor or cyst was reported. Common ultrasound signs of ovarian torsion are an enlarged adnexa, whirlpool sign, ovarian stromal edema with or without peripherally displaced antral follicles, and free fluid in the pelvis.^[4]^ Occasionally, an ovarian mass with absence or decrease in arterial flow in the twisted ovarian pedicle on color Doppler ultrasound can be helpful in differentiating ovarian torsion.^[4]^

The incidence of malignant ovarian torsion is < 2%, primarily due to the presence of peripheral inflammation, adhesion formation, or invasion in cases of ovarian cancer.^[2,5,6]^ Transvaginal ultrasound is an integral part of ovarian tumor screening and is associated with a higher risk of malignancy with abnormal morphology, including solid area or papillary projections from the cyst wall, increased volumes(>20 cm^3^ in premenopausal women and > 10 cm^3^ in postmenopausal women), cysts with septations, and ascites.^[7]^ Transvaginal ultrasound is often used as a screening tool for adnexal tumors and malignancies should be considered when measuring the size of an ovarian tumor that is heterogeneous with solid components or papillary excrescence.^[7]^ However, in cases of tumors with torsion, squeezed tumor containment may mimic malignant features in ultrasound images. Accurate preoperative diagnosis of ovarian cancer in these circumstances is challenging because the features are similar to those of benign tumors with torsion, especially when internal bleeding and emergent surgery are indicated.^[3]^

This report describes a rare case of ovarian torsion and ruptured cystadenocarcinoma in a postmenopausal woman with a retroperitoneal hematoma. The authors wish to attract readers to the tricks on preoperative diagnosis and surgical tips to optimize treatment strategies and improve survival rates.

## 2. Case presentation

**Figure 1.**
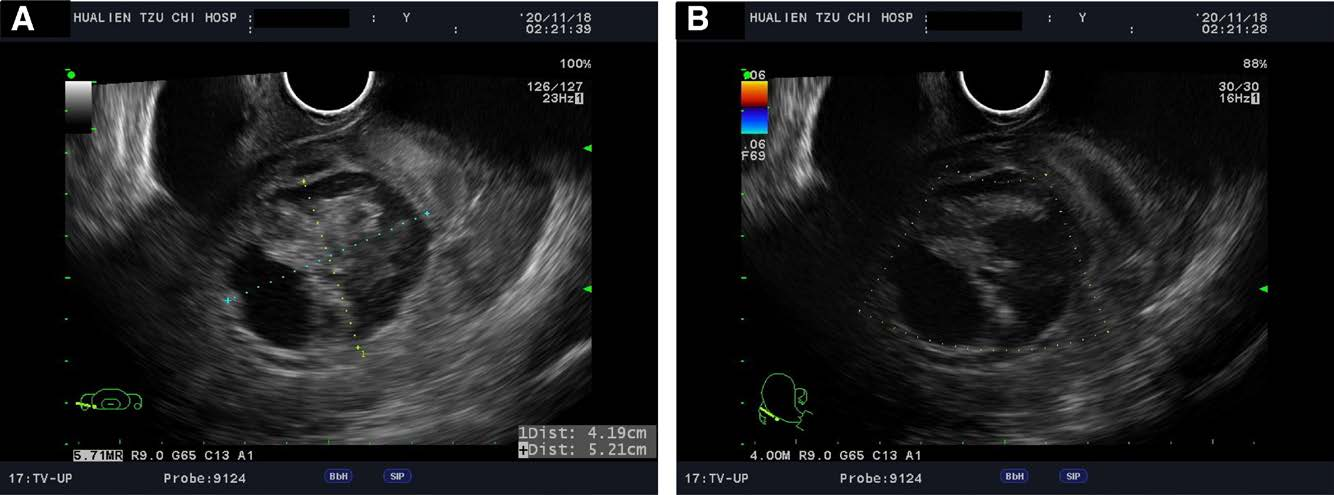
(A) Transvaginal ultrasound showed a heterogeneous right adnexal mass about 5 × 6 × 5 cm in size with soft tissue and fluid in content. (B) Absence of arterial flow on color Doppler within the adnexal tumor.

**Figure 2.**
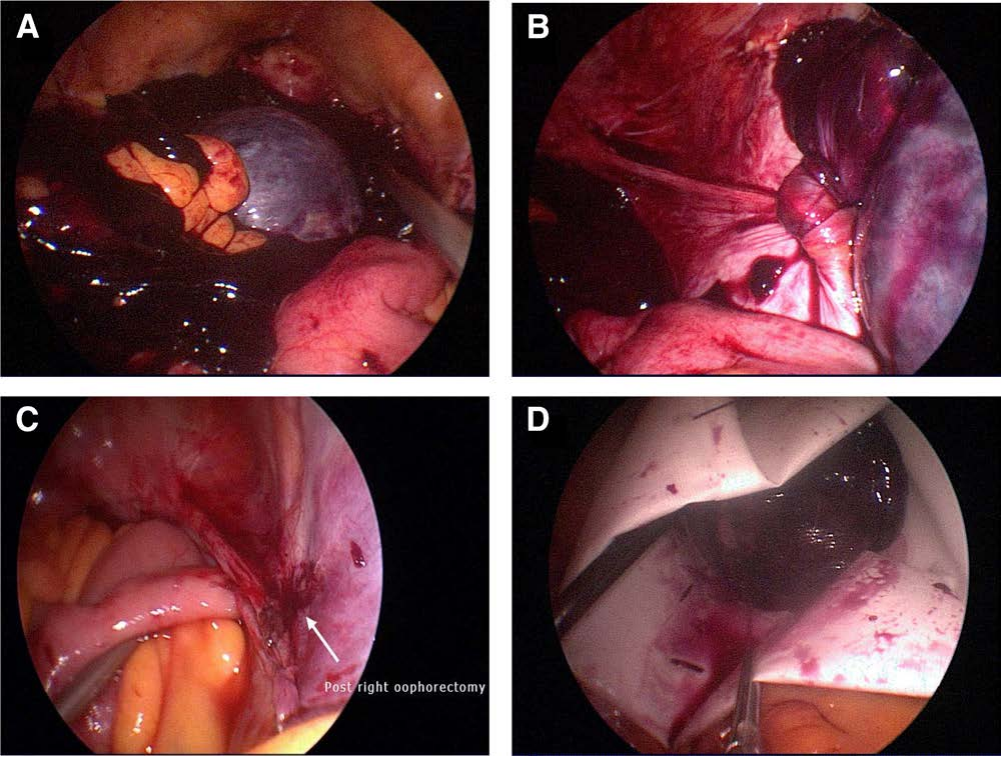
(A) Hemoperitoneum was noted during laparoscopic exploration. (B) Multiple torsions of the right ovarian tumor. (C) Right infundibulopelvic ligament retroperitoneal hematoma was found after tumor transection. (D) Right ovarian tumor was put in a tissue bag and removed by contained manual morcellation.

A 51-year-old woman, gravida 4, para 2, abortion 2, underwent laparoscopic total hysterectomy at 44 years of age due to leiomyoma. She presented to the emergency department with acute abdomen accompanied by nausea and vomiting at midnight. Physical examination revealed diffuse abdominal tenderness and rebound pain. A pelvic examination revealed severe lifting pain and right adnexal tenderness. Transvaginal ultrasound showed a right ovarian tumor about 5 × 6 × 5 cm in size with heterogeneous content. Absent ovarian blood flow was noted using color Doppler ultrasonographyx (Fig. 1), some soft tissue-like material and fluid were also present in the pelvic cavity. Laboratory test results including complete blood count, blood coagulation, liver function, renal function, and urine analysis were normal. Pelvic computed tomography revealed an 8-cm heterogeneous pelvic mass. Two days later, the test for serum tumor markers revealed normal levels of cancer antigen 125 (CA-125) at 12.7 U/mL (range 0–35 U/mL) and carbohydrate antigen 19–9 at 3.12 U/mL (range 0–35 U/mL). In the emergent diagnosis laparoscopy, a congested infarcted and ruptured right ovarian tumor was found twisted with 2.5 circles. Tumor bleeding, with a total of 900 mL of hemoperitoneum, was also noted during the operation (Fig. 2). The patient underwent right salpingo-oophorectomy with LigaSure. After tumor resection, continuous oozing and hematoma occurred in the infundibulopelvic ligament region, and we attempted packing with Surgicel but failed to stop the bleeding. The patient was successfully treated with hemoclips after identifying the position of the right ureter. The specimens were removed through the umbilical port site by contained manual morcellation with a tissue pouch.

**Figure 3.**
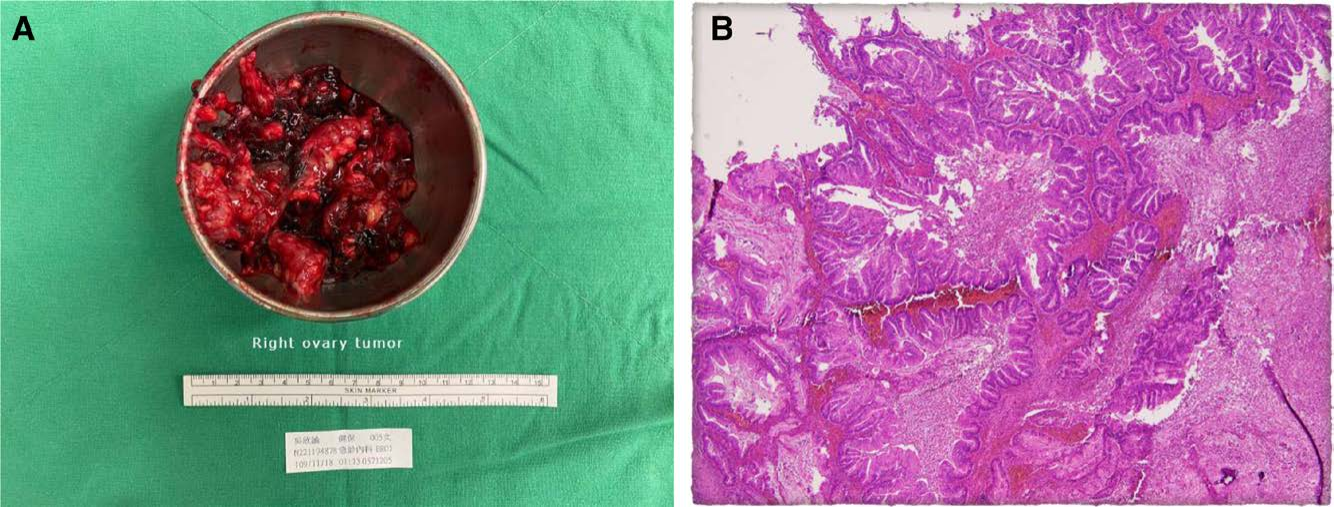
(A) Gelatinous ovarian tumor with blood clots coating after contained manual morcellation. (B) Complex glandular structures with mucinous epithelium and expansile stromal invasion were found under microscopy.

The gross appearance of the ovarian tumor was heterogeneous black gray-whitish with a marked gelatinous and blood clot coating (Fig. 3). Microscopically, it shows cyst-like lesions with gelatinous content and neoplastic glands lined by mucinous epithelium showing a variable degree of architectural complex, including stratification and tufting papillae, containing goblet cells, and presenting stromal invasion. Hemorrhages were also observed. Unexpectedly, histopathology revealed a mucinous cystadenocarcinoma of the right ovary, pT1c2.

On follow-up, we provided full informed consent to the patient and her husband, and the discussion included the final pathology, necessity of restaging, recurrence rate, and choice of adjuvant therapy. The patient and family were very worried about the dissemination of cancer cells because tumor rupture was found during the first emergent laparoscopic surgery. We also discuss the advantages and disadvantages of prophylactic hyperthermic intraperitoneal chemotherapy (HIPEC), including the lack of beneficial evidence when used in ruptured early stage ovarian cancer. Two weeks later, a subsequent laparoscopic staging surgery (left salpingo-oophorectomy, bilateral pelvic lymph node dissection, omentectomy, appendectomy) and prophylactic HIPEC with cisplatin 90 mg/m^2^ were performed. Histopathology of the resected specimens, including the left ovary, left fallopian tube, omentum, appendix, bilateral pelvic lymph node, and right IP ligament, showed no malignancy. Therefore, the final pathological stage of the ovarian cancer in this patient was pT1c2N0M0.

## 3. Outcome and follow-up

She has been regularly followed up every 3 months with transvaginal sonography for 4 years, including tumor marker once every 6 months and abdominal computed tomography once every year and no evidence of recurrence is being found.

## 4. Discussion

Spontaneous rupture of ovarian tumors is an uncommon, but significant event. This phenomenon can occur due to internal bleeding or increased pressure within the tumor and is often exacerbated by factors such as anticoagulant therapy or congestive heart failure.^[8]^ The occurrence of tumor rupture in this case was most likely due to torsion, as the patient did not have any related medical conditions. This is a very rare occurrence, and we would like to discuss some valuable clinical points. First, the diagnosis of a malignant ovarian tumor with torsion and rupture is very challenging and may not be accurately confirmed even during surgery, as the hemorrhagic content and ischemic swelling can mimic the morphology of a benign ovarian torsion, particularly when accompanied by internal bleeding. Although ovarian cancer with torsion is a rare event, this diagnosis should always be considered in our practice. Preoperative tumor marker tests and intraoperative frozen sections should be considered when ovarian malignancy is suspected. However, as in our case, the event occurred at 3 o’clock in the morning, and neither were available thereafter. This may delay the diagnosis of cancer prior to surgery.

Second, to resect an ovarian tumor with torsion, the ovarian tumor should be detected so that the vascular pedicles can be fully coagulated by electrocauterization devices before transection. However, only partial tumor detorsion was achieved in our case because of internal bleeding and the twists were very tight. Therefore, the pedicle of the infundibulopelvic ligament vessels contracts into the pelvic retroperitoneum, followed by bleeding and hematoma formation after tumor resection. Hemostasis with ligation or coagulation is indicated if compression does not work, and hemostasis for bleeding at the infundibulopelvic ligament site should be performed cautiously to avoid ureter injury. After exposure and identification of the ureteral position, ligation, electrocauterization, or hemoclip can effectively and safely stop bleeding from the vascular pedicles.^[9]^ In this case, we chose to use a hemoclip rather than electrocauterization for hemostasis to avoid thermal injury. In the subsequent staging surgery, we performed laparoscopic surgery instead of laparotomic surgery because of the early stage of cancer and the advantages of the laparoscopic approach.^[10]^ In consideration of the patient’s safety and efficacy, we recommended using in-bag morcellation^[11]^ or contained manual morcellation^[9]^ as in this reported case, that is, containing the specimen with a tissue bag before cutting the specimen in strips for every minimally invasive surgery. This approach prevents the retention of residual tissue fragments and further dissemination of malignant cells.^[9,10]^

Finally, a relapse rate of 9% to 29% has been reported for conservative treatment of early stage ovarian cancer.^[12,13]^ Spontaneous rupture of ovarian tumors, particularly cystadenocarcinomas, is a critical risk factor for recurrence^[14]^ and results in less favorable staging and prognosis for the patient.^[15,16]^ This is a nightmare for every 1 of all patients. Furthermore, the timing of tumor rupture significantly affects survival outcomes. Research indicates that patients whose tumors rupture before surgery have poorer survival rates than those whose tumors rupture during surgical manipulation. Specifically, patients with preoperative ruptures had a survival rate of 59%, whereas those with intraoperative ruptures had a survival rate of 85%.^[17]^ Several studies have shown the benefits of HIPEC in interval procedures and recurrent diseases in ovarian cancer.^[18,19]^ In addition to conventional intravenous chemotherapy, HIPEC is an alternative option that can be delivered at the time of cytoreductive surgery. HIPEC can improve the direct concentration of chemotherapy within the peritoneal cavity compared to the intravenous route, and may also reduce the systemic side effects related to intravenous chemotherapy or prolonged adjuvant intraperitoneal chemotherapy.^[20]^ Owing to the rupture of the cancer tumor, the patient was very worried about the risk of recurrence. The patient also resisted intravenous chemotherapy for recurrence. Therefore, prophylactic HIPEC was fully discussed with the patient and her family members. After explaining the advantages and disadvantages of HIPEC, she decided to receive prophylactic HIPEC in the subsequent surgery for better control of the disseminated cancer cells to kill and wash out residual cancer cells. She is now follow-up every 3 months at our outpatient clinic. No evidence of local recurrence was found during the follow-up examinations at 4 years. However, further studies are needed to investigate the benefits of HIPEC for treating ruptured early stage ovarian cancer.

## 5. Limitation

Due to the presence of only 1 case in this study, the generalizability is limited and should be recognized. There are no effective preoperative diagnostic and treatment guidelines for ruptured malignant ovarian tumors with torsion. Although the patient had a good prognosis after staging surgery concurrent with HIPEC, the benefit of HIPEC for ruptured early stage ovarian cancer is uncertain and further studies are needed to confirm.

## 6. Conclusion

Ovarian tumors presenting with torsion and internal bleeding can complicate the diagnosis of malignancy and should be considered in daily medical practice. Complete detorsion should be performed before tumor resection to prevent bleeding due to incomplete coagulation or ligation. Additionally, we recommend the use of contained morcellation with a tissue bag for specimen removal during minimally invasive surgery, to minimize the potential dissemination of cancer cells.

## Author contributions

**Conceptualization:** Chin-Tzu Tien, Mun-Kun Hong.

**Data curation:** Chin-Tzu Tien, Chiu-Hsuan Cheng, Mun-Kun Hong.

**Resources:** Mun-Kun Hong.

**Supervision:** Mun-Kun Hong.

**Writing – original draft:** Chin-Tzu Tien, Mun-Kun Hong.

**Writing – review & editing:** Chin-Tzu Tien, Mun-Kun Hong.
